# Das neurourologische Gutachten in der gesetzlichen Unfallversicherung: Konsentierte Empfehlungen zur Diagnostik und zur Bewertung der Minderung der Erwerbsfähigkeit

**DOI:** 10.1007/s00120-023-02039-y

**Published:** 2023-03-03

**Authors:** A. Wolff, N. Münstermann, J. Pretzer, A. Redecker, T. Jud, R. Böthig

**Affiliations:** 1 Abteilung Neuro-Urologie, BG-Unfallklinik Murnau, Professor-Küntscher-Str. 8, 82418 Murnau, Deutschland; 2 Marienhospital Herne, Herne, Deutschland; 3 BG Unfallkrankenhaus Berlin, Berlin, Deutschland; 4 Bergmannstrost BG Klinikum Halle, Halle, Deutschland; 5 AUVA Rehabilitationszentrum Häring, Bad Häring, Österreich; 6 BG Klinikum Hamburg, Hamburg, Deutschland

**Keywords:** Gutachterliche Diagnostik, Unfallverletzungen, Blasenfunktionsstörung, Darmfunktionsstörungen, Sexualfunktionsstörungen, Expert diagnosis, Accidents, occupational, Bladder dysfunction, Intestinal dysfunction, Sexual dysfunction

## Abstract

**Hintergrund:**

Bisherige Bewertungsvorgaben der Standardquellen für urologische Begutachtungen zeigen erhebliche Unterschiede in den empfohlenen Prozentangaben für die Einschätzung der Minderung der Erwerbsfähigkeit (MdE) für Unfallfolgeschäden auf neurourologischem Fachgebiet.

**Fragestellung:**

Es sollte für Begutachtungen im Rechtsbereich der Deutschen bzw. Österreichischen Gesetzlichen Unfallversicherung (www.dguv.de, www.auva.at) eine „Neufassung und Vereinheitlichung der MdE-Bewertungen neurourologischer Unfallfolgen (in Tabellenform) als Leitfaden/Manual“ erarbeitet werden.

**Material und Methoden:**

Innerhalb des Arbeitskreises Neurourologie der DMGP (Deutschsprachige Medizinische Gesellschaft für Paraplegiologie; www.dmgp.de) bildete sich eine Arbeitsgruppe aus Neurourologen aus Querschnittgelähmtenzentren verschiedener berufsgenossenschaftlicher (BG-)Kliniken. Zwischen 01/2017 und 09/2022 fanden insgesamt 7 Arbeitstagungen und 6 Videokonferenzen statt. Die Konsentierung der erarbeiteten Dokumente erfolgte durch formale Konsensusfindung im Rahmen eines nominalen Gruppenprozesses bzw. im Rahmen einer abschließenden Konsensuskonferenz.

**Ergebnisse:**

Es wurden die notwendigen Grundlagen für eine zielgerichtete, rechtssichere Diagnostik von Unfallfolgen auf neurourologischem Fachgebiet ausgearbeitet und basierend auf der Erfahrung langjähriger Gutachtertätigkeit eine „Matrix“ für eine einheitliche, abgestufte Einschätzung der Höhe der MdE auf (neuro)urologischem Fachgebiet bei gesicherten neurourologischen Unfallfolgen erstellt.

**Schlussfolgerung:**

Im Interesse der Gleichbehandlung aller Versicherten ist es von großer Bedeutung, eine einheitliche und nachvollziehbare Einschätzung der Höhe der MdE auf der Grundlage von „Tabellenwerten“ vorzunehmen, die die vorliegenden Erfahrungswerte widerspiegeln.

**Zusatzmaterial online:**

Die Online-Version dieses Beitrags (10.1007/s00120-023-02039-y) enthält zusätzliche Fragenkataloge.

## Abstract (en)

**Background:**

Previous assessment guidelines from standard sources for urologic expert opinions show considerable differences in the recommended percentages for the assessment of reduction of earning capacity (MdE) for accident sequelae in the neuro-urological specialty.

**Objectives:**

To develop a “revised and standardized version of the MdE assessments of neuro-urological accident sequelae (in tabular form) as a guideline/manual” for expert opinions in the legal area of the German and Austrian Statutory Accident Insurance (www.dguv.de, www.auva.at).

**Materials and methods:**

A working group of neuro-urologists from spinal cord injury centers of different BG (“Berufsgenossenschaft”) clinics was formed within the working group Neuro-Urology of DMGP (German-speaking Medical Society for Paraplegiology; www.dmgp.de). Between January 2017 and September 2022, a total of 7 working meetings and 2 video conferences were held. The consensus of the developed documents was reached by formal consensus finding in a nominal group process and in a final consensus conference.

**Results:**

The necessary bases for a targeted, legally sound diagnosis of accident consequences in the neuro-urological field were elaborated and, based on the experience of many years of expert opinion activity, a “matrix” for a uniform, graduated assessment of the level of reduction of earning capacity in the (neuro-)urological field in the case of confirmed neuro-urological accident consequences was created.

**Conclusion:**

In the interest of equal treatment of all insured persons, it is of great importance to make a uniform and comprehensible assessment of the amount of the MdE on the basis of “table values” that reflect the available empirical values.

Neurourologische Begutachtungen für die Gesetzliche Unfallversicherung setzen eine hohe Expertise in der Diagnostik neurourologischer Funktionsdefizite nach Unfallverletzungen und vertiefte Kenntnisse im Gutachtenwesen voraus. In der Vergangenheit zeigten Gutachten bei vergleichbaren Unfallfolgen erhebliche Unterschiede in der angewandten Diagnostik und der Höhe der empfohlenen Minderung der Erwerbsfähigkeit (MdE).

Eine Arbeitsgruppe aus erfahrenen Gutachtern hat Empfehlungen zur adäquaten Diagnostik sowie für eine einheitliche, abgestufte Einschätzung der Höhe der MdE im neurourologischen Fachgebiet bei gesicherten Unfallfolgen erarbeitet.

Die Motivation für die Erarbeitung der vorliegenden Empfehlungen ergab sich aus der Erkenntnis, dass die Höhe der empfohlenen MdE bei ähnlichen Unfallfolgen auch zwischen erfahrenen Gutachtern aus unterschiedlichen berufsgenossenschaftlicher (BG-)Kliniken bei Gutachten mit neurourologischer Fragestellung häufig erhebliche Unterschiede aufweist.

Bei der Orientierung an Bewertungsvorgaben bisheriger Standardquellen für urologische Begutachtungen [3, 5, 18, 20, 21, 23] zeigen sich zwischen verschiedenen Autoren ebenfalls weit auseinander liegende Prozentangaben für die empfohlene Einschätzung der MdE für Unfallfolgeschäden im neurourologischen Fachbereich. Diese bisherigen Standardquellen bieten einen erheblichen Interpretationsspielraum und damit keine einheitliche Bewertungsgrundlage. Darüber hinaus sind diese Quellen teilweise 10 bis 25 Jahre alt.

Die Standardquellen für urologische Begutachtungen bieten keine einheitliche Bewertungsgrundlage

Ziel war es daher, konsentierte Empfehlungen erfahrener neurourologischer Gutachter zur MdE-Einschätzung in einer neuen, nachvollziehbar begründeten „Matrix“ zusammenzufassen. Diese sollen als Orientierungshilfe und zur Erleichterung bei der Einschätzung der MdE in neurourologischen *(Zusatz-)*gutachten im Geltungsbereich der Gesetzlichen Unfallversicherung dienen.

Bei der gutachterlichen Beurteilung von neurourologischen Unfallfolgen sind neben den zu bewertenden Störungen der Funktion des unteren Harntrakts häufig auch mögliche Störungen der Sexualfunktion und der Darmfunktion zu bewerten. Die Bewertungen dieser Funktionsstörungen gehen daher ebenfalls in die „MdE-Matrix“ ein.

Voraussetzung für die korrekte gutachterliche Bewertung von neurourologischen Unfallfolgen ist eine zielführende und nachvollziehbare Diagnostik.

Durch eine adäquate Diagnostik ist eine auf die gutachterliche Fragestellung bezogene Objektivierung der Unfall- oder Krankheitsfolgen zu sichern [26]. Alle in die gutachterliche Bewertung einfließenden Funktionsminderungen müssen durch entsprechende diagnostische Maßnahmen im rechtlichen Sinne im „Vollbeweis“ gesichert werden [25]. In jedem Gutachten sollte stets eine maximale Annäherung an die Wahrheit erreicht werden, das heißt, es dürfen keine begründeten Zweifel mehr bestehen [6].

Für die Annahme, dass eine Gesundheitsstörung Folge einer Schädigung ist, genügt versorgungsrechtlich im Geltungsbereich der Gesetzlichen Unfallversicherung die „hinreichende“ Wahrscheinlichkeit des ursächlichen Zusammenhangs. Sie ist gegeben, wenn nach der medizinisch-wissenschaftlichen Lehrmeinung mehr für als gegen einen ursächlichen Zusammenhang spricht.

Neurourologische Unfallfolgen können nach Verletzungen des zentralen sowie des peripheren Nervensystems auftreten. Der begutachtende Neurourologe setzt sich schwerpunktmäßig mit Störungen nach Verletzungen des Rückenmarks und daraus resultierenden inkompletten oder kompletten Querschnittlähmungen, nach Schädel-Hirn-Verletzungen oder auch nach Beckenverletzungen mit peripheren Nervenläsionen auseinander. Zudem sind direkte Organverletzungen des Harntrakts und der Genitalien sowie deren funktionelle Folgen zu beurteilen.

Es handelt sich fast immer um funktionelle Beeinträchtigungen, die als Folge einer unfallbedingten Verletzung oder aber unfallunabhängig als Folge krankhafter Veränderungen auftreten können. Insofern ist bei fast jeder neurourologischen Begutachtung eine Zusammenhangsbewertung zwischen dem Unfallschaden und den diagnostisch gesicherten, funktionellen Defiziten vorzunehmen.

Komorbiditäten (Schadensanlagen und Vorerkrankungen) und mögliche Arzneimittelnebenwirkungen auf urologische Funktionen müssen erkannt und bewertet werden.

## Methoden

Ausgehend von oben skizzierter Fragestellung bildete sich innerhalb des Arbeitskreises Neurourologie der DMGP (Deutschsprachige Medizinische Gesellschaft für Paraplegiologie; www.dmgp.de) eine Arbeitsgruppe von Neurourologen aus Querschnittgelähmtenzentren verschiedener BG-Kliniken als auch der Abteilung für Neurourologie des Marien-Hospital Herne zur gemeinsamen „Erstellung eines Leitfadens zur gutachterlichen Beurteilung von neurourologischen Unfallfolgen“. Als Ziel wurde eine „Neufassung und Vereinheitlichung der MdE-Bewertungen neurourologischer Unfallfolgen (in Tabellenform) als Leitfaden/Manual“ geplant. Es ging ausdrücklich um Begutachtungen im Rechtsgebiet der Deutschen bzw. Österreichischen Gesetzlichen Unfallversicherung (www.dguv.de, www.auva.at).

In der Folge fanden seit Januar 2017 insgesamt 7 Arbeitstagungen und 6 Videokonferenzen mit Teilnehmern aus den BG-Kliniken Murnau, Halle (Saale), Berlin, Hamburg, Häring (AUVA) und der Neurourologischen Abteilung des Marienhospitals Herne statt.

In diesem Rahmen wurden die notwendigen diagnostischen Grundlagen der neurourologischen Begutachtung konsentiert, sowie letztendlich eine Matrix als Grundlage zur konkreten MdE-Bewertung erstellt und im Verlauf weiterentwickelt. Diese Matrix wurde seit September 2017 in konkreten BG-Begutachtungsfällen der Arbeitsgruppenmitglieder im klinischen Alltag angewendet, erprobt und somit evaluiert. Im Rahmen der Arbeitstagungen wurden die Erfahrungen der „Probe“-Anwender ausgewertet und diskutiert. Diese Erfahrungen bildeten die Grundlage für die weitere Ausarbeitung der Matrix durch formale Konsensusfindung im Rahmen eines Nominalen Gruppenprozesses [17].

Eine Videourodynamik ist notwendige Voraussetzung der gutachterlichen Diagnostik einer nLUTD

Darüber hinaus wurden im Rahmen der Arbeitstagungen die Grundsätze der notwendigen und zielgerichteten gutachterlichen Diagnostik von neurogenen Funktionsstörungen des unteren Harntraktes (neurogenic Lower Urinary Tract Dysfunction, nLUTD) sowie der neurogenen Sexualfunktionsstörungen und neurogenen Darmfunktionsstörungen diskutiert. Auch hier kam die Technik des nominalen Gruppenprozesses zur Konsensusfindung zur Anwendung. Die finale Version der Matrix und die empfohlenen diagnostischen Maßnahmen und Prozeduren fanden in der abschließenden Konsensuskonferenz vom 21.09.2022 einen „starken Konsens“ (Zustimmung von > 95 % der Teilnehmer).

## Ergebnisse

### Grundsätze der gutachterlichen Diagnostik von nLUTD

#### Anamnese

Am Beginn steht die Erhebung der allgemeinen Anamnese innerhalb und außerhalb des urologischen Fachgebietes *vor* dem Unfallereignis bzw. der anerkannten Berufserkrankung.

Im urologischen Bereich gehören dazu die Fragen nach:

- Miktionsverhalten (Miktionsfrequenz in 24 h, Nykturie, Pressmiktion, Restharngefühl, Harnnachträufeln, Inkontinenz),
- blasenspezifische Medikamente,
- Vorerkrankungen (z. B. benignen Prostatahyperplasie [BPH], Harnwegsinfektion, Tumoren),
- Voroperation (z. B. transurethrale Resektion der Prostata [TURP], Steinentfernungen, Tumorentfernung),
- Schwangerschaften und vaginale Entbindungen,
- urogenitale Verletzungen.

Im nicht-urologischen Bereich muss gefragt werden nach Erkrankungen, Verletzungen und Operationen, die eine (neurogene) Blasenfunktionsstörung verursachen könnten, z. B.:

- Wirbelsäulenveränderungen,
- Bandscheibenschäden,
- Myelomeningozele (Myelomeningozele [MMC], Spina bifida),
- Diabetes mellitus,
- multiple Sklerose,
- Morbus Parkinson,
- Operationen und Bestrahlungen an der Wirbelsäule, am/im Becken, im gynäkologischen Bereich,
- bestimmte Medikamente, insbesondere starke Analgetika und Medikamente aus dem neurologischen/psychiatrischen Fachbereich [14, 26].

Erleichtert wird die Erhebung der Anamnese, indem man der unfallverletzten bzw. erkrankten Person im Vorfeld der gutachterlichen Untersuchung einen Fragenkatalog zuschickt (s. Suppl. 1 als Zusatzmaterial online). Die beantworteten Fragen werden dann am Untersuchungstag vorgelegt und können für die Erstellung des Gutachtens genutzt werden.

Die persönlichen Angaben des Patienten sollten ergänzt werden durch die Auswertung von möglichst im Vorfeld der gutachterlichen Untersuchung verschickten Fragebögen/Protokollen:

- Trink‑/Miktions‑/Katheter‑/Inkontinenzprotokoll über mindestens 3 Tage [24],
- spezielle Fragebögen zur Blasenfunktion:
  - International Prostate Symptom Score (IPSS; [9]),
  - International Consultation on Incontinence Questionnaire-short form (ICIQ-SF; [7]);
- spezielle Fragebögen zur Darmfunktion:
  - Arbeitskreis Neurogene Darmfunktionsstörung (AND; [15]),
- NBD (Neurogenic Bowel Dysfunction Score; [12]),
- spezielle Fragebögen zur Sexualfunktionsstörung,
  - International Index of Erectile Function 5 (IIEF‑5; [8]),
  - Female Sexual Function Index (FSFI‑d; [2]).

Es folgt die Erhebung der speziellen urologischen Anamnese nach dem Unfallereignis bzw. seit Bestehen der anerkannten Berufserkrankung:

- Harnblasenentleerung bzw. Harnableitung (Via naturalis, transurethraler Dauerkatheter [DK], suprapubischer Katheter [SPK], intermittierender Katheterismus [13]), Dauer?
- Veränderungen wahrgenommen nach Entfernung des Katheters?
- Komplikationen (Makrohämaturie, Harnwegsinfekte, Harnverhaltung, Inkontinenz)?
- urologische Operationen und Operationen mit denkbarer Affektion des Urogenital- oder Nervensystems?

#### Untersuchungen

- Sensomotorische Funktion obere/untere Extremitäten:
  - Fußgänger ± Gehhilfe? Rollstuhlfahrer?
  - Schmerzen im Rahmen der Fortbewegung?
  - Arm- und Handfunktion? (Be- und Entkleiden, Selbstkatheterismus?)
- Urologischer Status:
  - Nierenlager, Abdomen, Leistenbruchpforten, Inspektion und Palpation des äußeren Genitals und des Anus, rektale Palpation.
  - Press- und Hustenmanöver im Liegen und im Stehen: Harninkontinenz?
  - Zysto‑/Rektozele? Analprolaps?
- Neurourologischer Status:
  - analer Sphinktertonus, Willkürkneifen des analen Sphinkters,
  - Reflexe (Bulbo-cavernosus-Reflex, Cremasterreflexe, Analreflex),
  - Sensibilität im Anogenitalbereich (S2–S5) testen.
  - Spitz-Stumpf-Diskrimination?
- Labordiagnostik:
  - Kreatinin, Harnstoff, Harnsäure, Cystatin C, errechnete glomeruläre Filtrationsrate (eGFR),
  - Elektrolyte,
  - kleines Blutbild,
  - Glukose, ggf. HbA1c,
  - ggf. prostataspezifisches Antigen (PSA),
  - Urinstatus, ggf. Urinsediment und Urinkultur.
- Sonographie:
  - Nieren, Harnblase, Prostata; ggf. Hoden/Nebenhoden und transrektaler Ultraschall (TRUS),
  - Restharn nach einer Uroflowmetrie.
- Uroflowmetrie
  - ggf. „double voiding“ bei Restharn.

#### Spezielle Untersuchungen, je nach Ergebnis der vorangegangenen Untersuchungen

- (Video)urodynamik (Zystomanometrie, Druck-Fluss-Studie, Beckenboden-Elektromyogramm [‑EMG], ggf. Eiswassertest; [16, 19]),
- retrograde Urethrographie,
- Urethrozystoskopie,
- PAD-Test (standardisierter 1‑h-PAD-Test; [11]),
- Nierenszintigraphie.

Eine qualifizierte Videourodynamik ist notwendige Voraussetzung der gutachterlichen Diagnostik einer nLUTD.

### Grundsätze der gutachterlichen Diagnostik von neurogenen Sexualfunktionsstörungen (nSFS)

#### Anamnese

Am Beginn steht die Erhebung der allgemeinen urologischen Anamnese mit Beleuchtung der sexuellen Situation vor dem Ereignis/Unfallgeschehen.

Die analytische Betrachtung der urologischen Vorgeschichte dient der Eruierung möglicher Faktoren, welche dazu geeignet sind, eine Sexualfunktionsstörung zu verursachen. Es folgt die Erhebung der speziellen urologischen Anamnese nach dem Unfall mit Fokussierung auf die Sexualität. Häufig ist hierzu auch ein intensives Aktenstudium mit Sammlung von Indizien erforderlich. Wenn möglich, ist ergänzend zur Eigenanamnese auch eine Partnerbefragung sinnvoll.

Im Zusammenhang mit Funktionsstörungen der Sexualität ist auf zwei wiederkehrende Beobachtungen hinzuweisen. Einerseits kann es sein, dass Funktionsstörungen im Urogenitalbereich von Unfallverletzten erst zeitlich verzögert wahrgenommen werden, da die unfallchirurgische Versorgung und Rehabilitation Priorität hat. Andererseits werden auch unfallunabhängig begründete urologische Störungen im Sinne einer Schadensanlage oder Vorerkrankung von Unfallverletzten aufgrund eines Kausalitätsbedürfnisses mit dem Unfall in Zusammenhang gebracht. Beide Phänomene sind vom Gutachter zu beachten und in seiner Bewertung entsprechend einzuordnen.

Im Rahmen der Anamnese muss eine Bestandsaufnahme der aktuellen Medikation mit genauer Tagesdosierung erfolgen [10]. Zu überprüfen sind Medikamente auf ihre potenziellen Nebenwirkungen im Bereich der Sexualität.

#### Weiterführende Anamnese zur weiblichen Sexualfunktionsstörung

- Eingetretene Veränderungen hinsichtlich des sexuellen Verlangens (Libido), der sexuellen Antwort und/oder der Scheidenbefeuchtung (Lubrikation),
- Qualität und Intensität der Wahrnehmung von Berührungen im Bereich des äußeren Genitales und eine möglicherweise gestörte Empfindlichkeit bzw. Schmerzhaftigkeit,
- Wahrnehmung des Orgasmus als (unverändert) positiv, reduziert oder ggf. Spastik auslösend oder schmerzhaft,
- begleitende, nicht vermeidbare Stuhl- und/oder Harninkontinenz,
- Zeichen einer autonomen Dysreflexie bei sexueller Aktivität,
- (unerfüllter) Kinderwunsch [22].

Die Verwendung des validierten Fragebogens FSFI‑d in deutscher Fassung [2] ermöglicht die weitere Differenzierung und Objektivierung der Sexualfunktionsstörung (Aufarbeitung der Bereiche Lust, Erregung, Lubrikation, Orgasmus, Befriedigung und Schmerz).

#### Weiterführende Anamnese zur männlichen Sexualfunktionsstörung

- Sexuelle Lust (Libido),
- Qualität und Intensität der Wahrnehmung von Berührungen im Bereich des äußeren Genitales und eine möglicherweise gestörte Empfindlichkeit bzw. Schmerzhaftigkeit,
- Fähigkeit zur Gliedsteife – mit einer möglichst präzisen Erfassung des erreichbaren Erektionsgrades (E0 bis E5), der Stabilität bzw. Dauer der erreichbaren Erektion sowie der Fähigkeit zur Penetration,
- Resultat einer ggf. bereits probatorischen Einnahme eines Phosphodiesterase (PDE)-5-Hemmers,
- Vorhandensein oder Fehlen nächtlicher oder morgendlicher Spontanerektionen,
- Erreichbarkeit von Samenerguss und Orgasmus,
- Quantität und Qualität des Ejakulats und der Ejakulation,
- mögliches Auftreten einer frühzeitigen Ejakulation (Ejaculatio praecox) und einer retrograden Ejakulation,
- Wahrnehmung des Orgasmus als (unverändert) positiv, reduziert oder ggf. Spastik auslösend oder schmerzhaft,
- zusätzliche Stuhl- und/oder Harninkontinenz,
- Zeichen einer autonomen Dysreflexie bei sexueller Aktivität,
- (unerfüllter) Kinderwunsch.

Unterstützend ist die Verwendung des validierten Fragebogens zur erektilen Funktion (IIEF‑5; [8]) sinnvoll.

#### Untersuchungsverfahren

Bei der körperlichen Untersuchung ist ein differenzierter Genitalstatus – vorzugsweise in Steinschnittlage – zu erheben. Hierbei müssen Befunde unfallunabhängiger Vorschäden oder Erkrankungen differenziert erfasst werden. Abzuklären ist auch das Vorliegen eines Deszensus oder Anal- bzw. Rektumprolapses.

Der neurourologische Status beinhaltet die Überprüfung der Sensibilität sowie die Erhebung des Reflexstatus, wobei alle neurovegetativen Reflexe im Anogenitalbereich zu überprüfen sind (s. Diagnostik der nLUTD).

Bei der Frau kann durch vaginale Palpation ein ggf. berichteter Introitus- oder Vaginalschmerz reproduziert und gesichert werden, so z. B. durch transvaginale Palpation der Spina ischiadica links und rechts (Druck auf den N. pudendus).

An die körperliche Untersuchung schließt sich die Sonographie des unteren Harntrakts an. Beim Mann sind das äußere Genitale und die Prostata (transrektal) sonographisch zu untersuchen.

Eine Farbduplexsonographie der Beckengefäße kann bei speziellen Fragestellungen zur Perfusion der Genitalorgane erfolgen.

Durch einen Schwellkörperinjektionstest (SKIT) mit Prostaglandin inklusive einer farbkodierten Dopplersonographie der penilen Gefäße kann eine vaskuläre Ursache der Erektionsstörung erfasst werden.

Bei speziellen Fragestellungen stehen folgende elektrophysiologische Untersuchungsmethoden (meist im Rahmen einer neurologischen Zusatzbegutachtung) zur Verfügung:

- EMG des M. sphincter ani externus (z. B. i. R. d. Urodynamik),
- Messung des Temperatur- und Vibrationsempfindens,
- elektrophysiologische Bestimmung der motorischen Pudenduslatenzzeit (PNTML) transanal mittels St. Marks-Fingerelektrode,
- Messung der somatosensorisch evozierten Potenziale (SSEP) zur Prüfung der Afferenz des N. pudendus,
- Aufzeichnung der genitalen sympathischen Hautantwort (GSHA),
- Bulbocavernosus-Reflex-Latenzzeitmessung.

Bei der Messung der PNTML und der SSEP ist unbedingt zu beachten, dass beide Messtechniken insbesondere die schnellleitenden Nervenfasern messen. Eine normale pudendale Latenz schließt das Vorliegen eines Schmerzsyndroms oder auch einer Störung der Sexualfunktion auf der Basis einer Pudendusschädigung nicht aus.

Die Anwendung der *nächtlichen Tumeszenz- und Rigiditätsmessung (Rigi-Scan)* kann beim Mann die Differenzierung zwischen organischer und psychischer Ursache einer Erektionsstörung unterstützen. Die Störanfälligkeit des Verfahrens bzw. die Manipulationsmöglichkeit bei der Durchführung und nicht zuletzt die mangelnde Verfügbarkeit eines Messgerätes schränken die Anwendung dieser Untersuchung ein.

#### Labordiagnostik

- Blutbild, Leberwerte, Nierenretentionswerte, Schilddrüsenwerte, Lipiddiagnostik, Blutzucker und HbA1c, beim Mann PSA,
- Hormonbestimmung: FSH, LH, Testosteron gesamt und frei, sexualhormonbindendes Globulin (SHBG), Prolaktin, Estradiol,
- Urindiagnostik,
- Spermiogramm (nach WHO) bei der Abklärung der Fertilität.

#### Weiterführende bildgebende Diagnostikmöglichkeiten

- Beckenübersicht,
- CT (Abdomen, Becken),
- Angio-CT Becken,
- MRT (Abdomen, Becken),
- Miktionszystourethrogramm (MCU), evtl. i. R. der Videourodynamik,
- Kavernosometrie.

Die nur sehr selten angezeigte Kavernosometrie kann Strukturschäden der Schwellkörper bzw. eine vaskuläre Problematik nachweisen.

Eine urodynamisch gesicherte unfallbedingte nLUTD stützt Annahme eines Unfallzusammenhangs der nSFS

Bei Hinweisen auf über das Verletzungsmuster hinausgehende neurologische und/oder psychiatrische Störungen sollte eine fachärztliche Vorstellung mit weiterführender Diagnostik erfolgen.

Ist das Verletzungsmuster des Unfallschadens dazu geeignet, Störungen der Sexualität zu begründen, und fehlen Vorerkrankungen und Schadensanlagen, ist die Objektivierung dieser Störungen durch Anamnese, körperliche Untersuchung, Sonographie und Labor oft bereits ausreichend.

Eine urodynamisch gesicherte unfallbedingte neurogene Funktionsstörung des unteren Harntrakts (nLUTD) stützt die Annahme eines Unfallzusammenhangs der Sexualfunktionsstörung.

### Grundsätze der gutachterlichen Diagnostik von neurogenen Darmfunktionsstörungen (nDFS)

Die Diagnostik der nDFS unterscheidet grundsätzlich zwischen den Maßnahmen der Basisdiagnostik, einer erweiterten Diagnostik und weiterführenden diagnostischen Maßnahmen bei speziellen Fragestellungen und wurde ausführlich in der Leitlinie Neurogene Darmfunktionsstörung bei Querschnittlähmung (Langfassung), AWMF-Register 179-004 [15] beschrieben. Daher kann an dieser Stelle auf die genannte Leitlinie explizit verwiesen werden.

### Die Bewertungsmatrix

**Figure 1:**
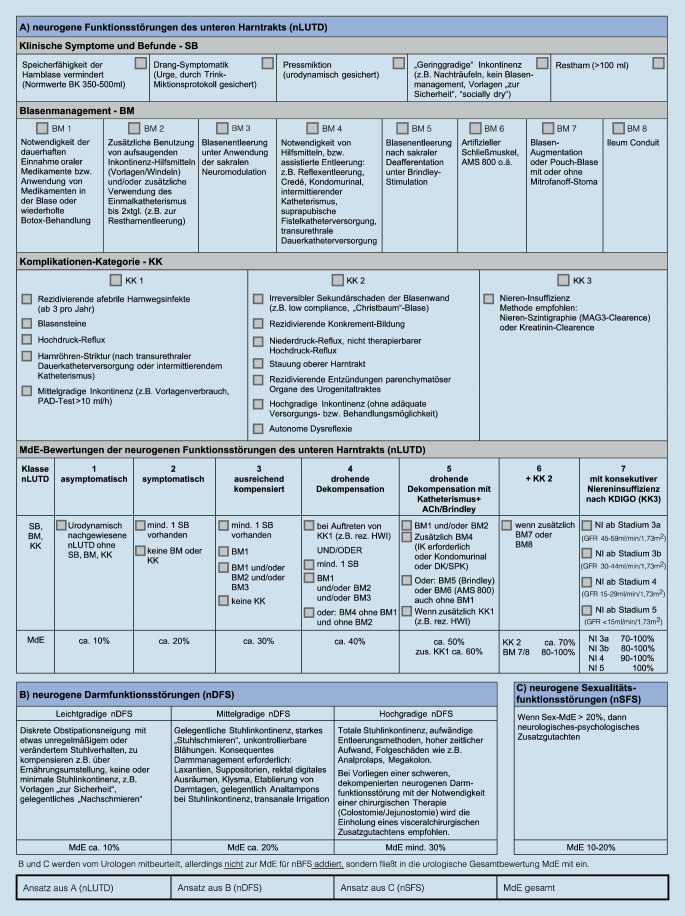

Die Matrix dient als wichtiges Werkzeug zur individuellen Bewertung der neurourologischen MdE (Abb. 1). Der Gutachter erhält nach gründlicher und adäquater Diagnostik durch die vorgegebene Bewertungsstruktur Anhaltspunkte zur einheitlichen und nachvollziehbaren Einordnung der zu beurteilenden Funktionsstörungen. Die so dokumentierte Einschätzung kann als Beleg zur Nachweisführung dienen.

## Diskussion

Der ärztliche Gutachter muss über die notwendige medizinische/sozialmedizinische Fach- und Sachkompetenz verfügen und die rechtlichen Grundlagen der Begutachtung kennen. Die gutachterliche Bewertung des medizinischen Sachverhalts muss sich nach dem – kritisch reflektierten – aktuellen Kenntnisstand in der medizinischen Wissenschaft richten. Abweichungen hiervon sind zwar im Einzelfall möglich, aber besonders zu kennzeichnen und eingehend zu begründen.

Der ärztliche Gutachter muss die medizinische/sozialmedizinische Fach- und Sachkompetenz haben

Für den ärztlichen Gutachter ist es zudem zwingend notwendig, sich mit den speziellen Rechtsgrundlagen der Begutachtung bzw. seines konkreten Gutachtenauftrags zu beschäftigen. Er muss die wesentlichen Rahmenbedingungen und die Unterschiede zwischen verschiedenen Versicherungs- bzw. Rechtsbereichen kennen. So existieren z. T. deutliche Unterschiede hinsichtlich der Anerkennungs- und Bewertungsmaßstäbe für die Feststellung von Funktionsstörungen in verschiedenen Rechts- bzw. Versicherungsbereichen, unterschiedliche Kausalitätsbegriffe im Straf‑, Sozial‑, Verwaltungs- und Haftungsrecht sowie verschiedene Regeln zu Beweismaß und -last [26]. Der Gutachter muss auch die häufig mit dem allgemeinen Sprachgebrauch nicht übereinstimmenden Definitionen in der juristischen Terminologie kennen. Dies gilt z. B. für Begriffe wie Minderung der Erwerbsfähigkeit (MdE), Grad der Behinderung bzw. der Schädigungsfolgen (GdB bzw. GdS), Berufs- und Erwerbsunfähigkeit, Berufskrankheit, teilweise und volle Erwerbsminderung, Arbeitsunfähigkeit, wesentliche Bedingung, leichte, mittelschwere oder schwere Arbeit, körperliche und geistige Behinderung bei Kindern usw. [1].

Die Berufsgenossenschaften und Unfallkassen als Träger der gesetzlichen Unfallversicherung verlangen im Rahmen der medizinischen Begutachtung ein besonderes Beweismaß. Die Verursachung eines Gesundheitserstschadens durch ein Unfallereignis oder eine schädigende (berufliche) Einwirkung wird als haftungsbegründende Kausalität bezeichnet. Hiermit tritt der „Versicherungsfall“ ein. Dies entscheidet die Verwaltung der zuständigen Berufsgenossenschaft auf der Grundlage der ihnen vorliegenden Unterlagen (Unfallmeldung, eigene Ermittlungen etc.).

**Figure 2:**
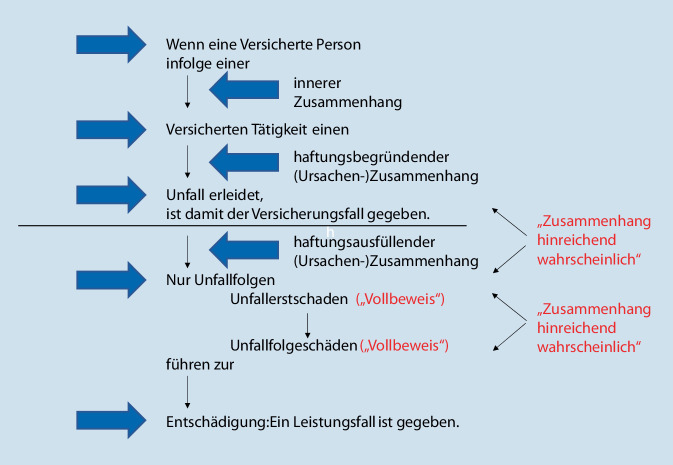

Die haftungsausfüllende Kausalität hingegen bezeichnet die gesundheitlichen Folgen des Arbeitsunfalls bzw. der Berufskrankheit, die zu einer Entschädigung führen („Leistungsfall“). Während sowohl der Gesundheitserstschaden als auch die funktionellen gesundheitlichen Folgen des Gesundheitserstschadens als Unfallfolgen gutachterlich im „Vollbeweis“ nachgewiesen werden müssen, reicht für die kausale Verknüpfung zwischen Unfallereignis bzw. schädigender Einwirkung und Gesundheitsschäden bzw. Funktionsbeeinträchtigungen die „hinreichende (einfache) Wahrscheinlichkeit“, d. h. nach ärztlicher Erkenntnis muss entsprechend der herrschenden medizinischen Lehrmeinung mehr für als gegen die Annahme eines Ursachenzusammenhangs sprechen, d. h. der haftungsausfüllende Zusammenhang ist gegeben, wenn die im „Vollbeweis“ gesicherten Gesundheitsschäden „rechtlich wesentlich“ durch den anerkannten Arbeitsunfall verursacht wurden. Der Beweismaßstab der „hinreichenden Wahrscheinlichkeit“ gilt auch für den Ursachenzusammenhang zwischen Gesundheitserstschaden und möglichen Gesundheitsfolgeschäden (Abb. 2).

Der „Vollbeweis“ bezeichnet nach einer Formulierung des Bundesgerichtshofes einen „für das praktische Leben brauchbaren Grad an Wahrscheinlichkeit, der verbleibende Zweifel zurücktreten lässt, ohne sie völlig auszuschließen“ (BGH v. 17.02.1970 – III ZR 139/67). Der Auftraggeber muss also durch die Beweisführung des Gutachtens zu einer subjektiven Gewissheit gelangen können.

Für die Frage der Kausalität, d. h. für die gutachterliche Überprüfung von Ursache-Wirkungs-Beziehungen und die Unterscheidung zwischen „ereigniskausalen“ und nicht auf das angeschuldigte Ereignis zurückzuführenden Schädigungen oder Funktionsbeeinträchtigungen gilt im Rechtsgebiet der Gesetzlichen Unfallversicherung die „Kausallehre von der wesentlichen Bedingung“. Danach muss der Gutachter neben dem angeschuldigten Schädigungsereignis (z. B. Unfall) auch konkurrierende Kausalitäten (z. B. Vorerkrankungen oder Schadensanlagen) berücksichtigen und in ihrer Bedeutung für den Schadenseintritt qualitativ bewerten. Eine rechnerische Gewichtung der einzelnen möglichen Ursachen soll jedoch nicht vorgenommen werden. Kommt einer der möglichen Ursachen (Unfallereignis oder Vorerkrankung) eine „überragende“ Bedeutung für den Kausalverlauf zu, drängt diese die jeweils anderen möglichen Ursachen in den Hintergrund und ist somit die alleinige „rechtlich wesentliche Ursache“. Dabei müssen im Rechtsgebiet der Gesetzlichen Unfallversicherung die versicherten bzw. entschädigungspflichtigen Ursachen im Vergleich zu den anderen möglichen Ursachen nicht „gleichwertig“ oder doch wenigstens „annähernd gleichwertig“ zum Schaden beigetragen haben. Somit reicht eine wesentliche Mitverursachung der Gesundheitsschädigung durch einen versicherten Unfall bzw. eine versicherte schädigende Einwirkung aus, um die volle Entschädigungsleistung zu erhalten („Alles-oder-nichts-Prinzip“). Abzugrenzen ist lediglich der Fall einer so schwerwiegenden Vorerkrankung bzw. Krankheitsanlage, dass auch Belastungen des alltäglichen Lebens den gleichen Schaden in etwa derselben Zeit und in etwa demselben Umfang hätten herbeiführen können. In diesem Fall träte der Unfall in seiner rechtlichen Bedeutung zurück und würde keine rechtlich wesentliche Bedingung mehr darstellen („Gelegenheitsursache“). Wird also das Unfallereignis als rechtlich wesentliche Ursache des Gesundheitsschadens eingeschätzt, besteht ein Leistungsanspruch für den gesamten Gesundheitsschaden (einschließlich möglicher Vorerkrankungen und Schadensanlagen).

Die MdE bezeichnet Umfang verminderter Arbeitsmöglichkeiten auf gesamtem Gebiet des Erwerbslebens

Im Rechtsgebiet der Gesetzlichen Unfallversicherung ist der Maßstab für die Bewertung von Gesundheitsstörungen die Höhe der MdE. Sie bezeichnet den Umfang der verminderten Arbeitsmöglichkeiten auf dem gesamten Gebiet des Erwerbslebens durch die Beeinträchtigung des körperlichen und psychischen Leistungsvermögens infolge eines Arbeitsunfalls oder einer Berufskrankheit. Im Interesse der Gleichbehandlung aller Versicherten wird hierzu auf Erfahrungswerte, tabellarisch dargestellt in der „maßgeblichen Rentenliteratur“, zurückgegriffen.

Wenn unterschiedliche, sich wechselseitig beeinflussende Funktionsbeeinträchtigungen in ihren Auswirkungen auf die Erwerbsfähigkeit zu beurteilen sind, wird die Gesamt-MdE für ein konkretes Unfallereignis integrierend gebildet.

Grundsätzlich wird eine abstrakte Schadensbemessung ohne Berücksichtigung der konkreten beruflichen Tätigkeit vorgenommen.

Vor dem Hintergrund dieser Besonderheiten der BG-Begutachtung wurde ein „Leitfaden zur gutachterlichen Beurteilung von neurourologischen Unfallfolgen auf dem Gebiet der Gesetzlichen Unfallversicherung“ erarbeitet und konsentiert. Beteiligt waren erfahrene Neurourologen aus verschiedenen BG-Kliniken (bzw. dem AUVA-Rehabilitationszentrum Häring bei Innsbruck) sowie der Abteilung für Neurourologie des Marien-Hospital Herne.

Besonderer Wert wurde darauf gelegt, den speziellen Anforderungen an die Begutachtung im Rechtsbereich der Gesetzlichen Unfallversicherung zu entsprechen. Die Notwendigkeit, sowohl den Unfallerstschaden als auch Unfallfolgeschäden im Vollbeweis zu sichern, kann nicht dazu führen, dass bei jedem zu begutachtenden Fall alle zur Verfügung stehenden diagnostischen Maßnahmen angewendet werden müssen. Vielmehr muss der Gutachter, auch in Kenntnis der Unfallfolgen auf anderen Fachgebieten (Neurologie!), auf der Basis einer sinnvollen, zielgerichteten Diagnostik zu einer Beurteilung gelangen, die im rechtlichen Sinne des Vollbeweises „möglichen Zweifeln Schweigen gebietet“. Die dazu notwendigen diagnostischen Schritte sind individuell durchaus unterschiedlich und wurden durch die Arbeitsgruppe diskutiert und konsentiert. Es ist allerdings festzuhalten, dass eine qualifizierte Video-Urodynamik eine notwendige Voraussetzung der gutachterlichen Beurteilung der nLUTD darstellt. Auch wenn bei vielen klinischen Fragestellungen eine Urodynamik mit gleichzeitiger Sonographie des Harntraktes diagnostisch ausreichend sein kann, ist bei gutachterlichen Fragestellungen zumindest eine einmalige videourodynamische Abklärung zu fordern. Larvierte, klinisch bisher nicht auffällige Störungen des unteren Harntrakts sind nach Detektion durch eine videourodynamische Untersuchung in die Bewertung mit einzubeziehen.

Ein Schwerpunkt der Arbeit lag auf der Entwicklung einer neuen, einheitlichen Orientierungshilfe zur MdE-Bewertung neurourologischer Funktionseinschränkungen, da die bisher vorliegende Vielzahl an Quellen zur MdE-Bewertung mit teils stark divergierenden Prozentangaben nicht mehr konstruktiv zur Orientierung und Entscheidungsfindung – v. a. bei weniger erfahrenen Gutachtern – beiträgt.

Durch die hier vorgestellte Matrix zur strukturierten Einschätzung der Höhe der MdE steht nun, im Vergleich zu vorangegangenen Veröffentlichungen, eine innovative und anwenderfreundliche Orientierungshilfe zur Verfügung, die im Interesse der Gleichbehandlung aller Versicherten eine präzise Entscheidungshilfe zur individuellen MdE-Bewertung neurourologischer Unfallfolgen bietet.

Es sei ausdrücklich betont, dass die vorliegende Matrix keinesfalls einen „mathematischen Algorithmus“ darstellt, der schematisch für jeden Unfallverletzten eine MdE „anzeigt“. Vielmehr handelt es sich um Anhaltspunkte für eine höchst individuelle gutachterliche Einschätzung, die – nach Auffassung der Autoren – im Sinne einer einheitlichen Bewertungsrichtlinie allen zukünftigen neurourologischen Begutachtungsfällen zugrunde gelegt werden sollte. Die Anwendung der Matrix trägt somit zu einer besseren Nachvollziehbarkeit der MdE-Einschätzung bei und verringert dadurch zeit- und kostenintensive Zweit- bzw. Nachbegutachtungen.

## Fazit für die Praxis

- Ausgehend von den Besonderheiten der Begutachtung im Rechtsbereich der Gesetzlichen Unfallversicherung werden konkrete Empfehlungen für eine zielführende und nachvollziehbare, rechtssichere, neurourologische, gutachterliche Diagnostik gegeben, mit der typische unfallbedingte Funktionsdefizite auf neurourologischem Fachgebiet (im Vollbeweis) gesichert werden können.
- Es wird eine Matrix vorgestellt zur einheitlichen, abgestuften Einschätzung der Höhe der Minderung der Erwerbsfähigkeit (MdE) auf (neuro)urologischem Fachgebiet bei gesicherten neurourologischen Unfallfolgen.
- Dabei werden neben Folgen neurogener Blasenfunktionsstörungen auch Folgen neurogener Darmfunktionsstörungen und Folgen neurogener Sexualfunktionsstörungen eingeschätzt.
- Diese Matrix wurde von erfahrenen neurourologischen Gutachtern erarbeitet und evaluiert.

## Supplementary Information





