# Supplementary material for: The neuro-urological expert opinion in statutory accident insurance: Consensual recommendations for diagnostics and for the assessment of reduction of earning capacity
Source: Urologie. 2023 Mar 3;62(3):229–40. [Article in German] doi: 10.1007/s00120-023-02039-y (PMC9998578; doi:10.1007/s00120-023-02039-y)
Supplement: Supplementary file 1 [file 120_2023_2039_MOESM1_ESM.pdf]

Matrix zur strukturierten Bewertung nLUTD/nDFS/nSFS und einheitlichen Einschätzung der Gesamt-MdE

A) neurogene Funktionsstörungen des unteren Harntrakts (nLUTD)

Klinische Symptome und Befunde - SB

Speicherfähigkeit der Harnblase vermindert (Normwerte BK 350-500ml) ☐	Drang-Symptomatik (Urge, durch Trink-Miktionsprotokoll gesichert) ☐	Pressmiktion (urodynamisch gesichert) ☐	„Geringgradige“ Inkontinenz (z.B. Nachträufeln, kein Blasen-management, Vorlagen „zur Sicherheit“, “socially dry“) ☐	Restharn (>100 ml) ☐

Blasenmanagement - BM

☐ BM 1	☐ BM 2	☐ BM 3	☐ BM 4	☐ BM 5	☐ BM 6	☐ BM 7	☐ BM 8
Notwendigkeit der dauerhaften Einnahme oraler Medikamente bzw. Anwendung von Medikamenten in der Blase oder wiederholte Botox-Behandlung	Zusätzliche Benutzung von aufsaugenden Inkontinenz-Hilfsmitteln (Vorlagen/Windeln) und/oder zusätzliche Verwendung des Einmalkatheterismus bis 2xtgl. (z.B. zur Restharnentleerung)	Blasenentleerung unter Anwendung der sakralen Neuromodulation	Notwendigkeit von Hilfsmitteln, bzw. assistierte Entleerung: z.B. Reflexentleerung, Credé, Kondomurinal, intermittierender Katheterismus, suprapubische Fistelkatheterversorgung, transurethrale Dauerkatheterversorgung	Blasenentleerung nach sakraler Deafferentation unter Brindley-Stimulation	Artifizieller Schließmuskel, AMS 800 o.ä.	Blasen-Augmentation oder Pouch-Blase mit oder ohne Mitrofanoff-Stoma	Ileum Conduit

Komplikationen-Kategorie - KK

☐ KK 1	☐ KK 2	☐ KK 3
☐ Rezidivierende afebrile Harnwegsinfekte (ab 3 pro Jahr)<br>☐ Blasensteine<br>☐ Hochdruck-Reflux<br>☐ Harnröhren-Striktur (nach transurethraler Dauerkatheterversorgung oder intermittierendem Katheterismus)<br>☐ Mittelgradige Inkontinenz (z.B. Vorlagenverbrauch, PAD-Test >10 ml/h)	☐ Irreversibler Sekundärschaden der Blasenwand (z.B. low compliance, „Christbaum“-Blase)<br>☐ Rezidivierende Konkrement-Bildung<br>☐ Niederdruck-Reflux, nicht therapierbarer Hochdruck-Reflux<br>☐ Stauung oberer Harntrakt<br>☐ Rezidivierende Entzündungen parenchymatöser Organe des Urogenitaltraktes<br>☐ Hochgradige Inkontinenz (ohne adäquate Versorgungs- bzw. Behandlungsmöglichkeit)<br>☐ Autonome Dysreflexie	☐ Nieren-Insuffizienz<br>Methode empfohlen:<br>Nieren-Szintigraphie (MAG3-Clearence) oder Kreatinin-Clearence

MdE-Bewertungen der neurogenen Funktionsstörungen des unteren Harntrakts (nLUTD)

**Klasse nLUTD**	**1 asymptomatisch**	**2 symptomatisch**	**3 ausreichend kompensiert**	**4 drohende Dekompensation**	**5 drohende Dekompensation mit Katheterismus+ ACh/Brindley**	**6 + KK 2**	**7 mit konsekutiver Niereninsuffizienz nach KDIGO (KK3)**
SB, BM, KK	☐ Urodynamisch nachgewiesene nLUTD ohne SB, BM, KK	☐ mind. 1 SB vorhanden<br>☐ keine BM oder KK	☐ mind. 1 SB vorhanden<br>☐ BM1<br>☐ BM1 und/oder BM2 und/oder BM3<br>☐ keine KK	☐ bei Auftreten von KK1 (z.B. rez. HWI)<br>UND/ODER<br>☐ mind. 1 SB<br>☐ BM1 und/oder BM2 und/oder BM3<br>☐ oder: BM4 ohne BM1 und ohne BM2	☐ BM1 und/oder BM2<br>☐ Zusätzlich BM4 (IK erforderlich oder Kondomurinal oder DK/SPK)<br>☐ Oder: BM5 (Brindley) oder BM6 (AMS 800) auch ohne BM1<br>☐ Wenn zusätzlich KK1 (z.B. rez. HWI)	☐ wenn zusätzlich BM7 oder BM8	☐ NI ab Stadium 3a (GFR 45-59ml/min/1,73m^2)<br>☐ NI ab Stadium 3b (GFR 30-44ml/min/1,73m^2)<br>☐ NI ab Stadium 4 (GFR 15-29ml/min/1,73m^2)<br>☐ NI ab Stadium 5 (GFR <15ml/min/1,73m^2)
MdE	ca. 10%	ca. 20%	ca. 30%	ca. 40%	ca. 50%<br>zus. KK1 ca. 60%	KK 2 ca. 70%<br>BM 7/8 80-100%	NI 3a 70-100%<br>NI 3b 80-100%<br>NI 4 90-100%<br>NI 5 100%

B) neurogene Darmfunktionsstörungen (nDFS)

Leichtgradige nDFS	Mittelgradige nDFS	Hochgradige nDFS
Diskrete Obstipationsneigung mit etwas unregelmäßigem oder verändertem Stuhlverhalten, zu kompensieren z.B. über Ernährungsumstellung, keine oder minimale Stuhlinkontinenz, z.B. Vorlagen „zur Sicherheit“, gelegentliches „Nachschmieren“	Gelegentliche Stuhlinkontinenz, starkes „Stuhlschmieren“, unkontrollierbare Blähungen. Konsequentes Darmmanagement erforderlich: Laxantien, Suppositorien, rektal digitales Ausräumen, Klysma, Etablierung von Darmtagen, gelegentlich Analtampons bei Stuhlinkontinenz, transanale Irrigation	Totale Stuhlinkontinenz, aufwändige Entleerungsmethoden, hoher zeitlicher Aufwand, Folgeschäden wie z.B. Analprolaps, Megakolon.<br>Bei Vorliegen einer schweren, dekompenierten neurogenen Darm-funktionsstörung mit der Notwendigkeit einer chirurgischen Therapie (Colostomie/Jejunostomie) wird die Einholung eines visceralchirurgischen Zusatzgutachtens empfohlen.
MdE ca. 10%	MdE ca. 20%	MdE mind. 30%

C) neurogene Sexualitäts-funktionsstörungen (nSFS)

Wenn Sex-MdE > 20%, dann neurologisches-psychologisches Zusatzgutachten
MdE 10-20%

B und C werden vom Urologen mitbeurteilt, allerdings nicht zur MdE für nBFS addiert, sondern fließt in die urologische Gesamtbewertung MdE mit ein.

Ansatz aus A (nLUTD)	Ansatz aus B (nDFS)	Ansatz aus C (nSFS)	MdE gesamt
