# Supplementary material for: The neuro-urological expert opinion in statutory accident insurance: Consensual recommendations for diagnostics and for the assessment of reduction of earning capacity
Source: Urologie. 2023 Mar 3;62(3):229–40. [Article in German] doi: 10.1007/s00120-023-02039-y (PMC9998578; doi:10.1007/s00120-023-02039-y)
Supplement: Supplementary file 2 [file 120_2023_2039_MOESM2_ESM.pdf]

Gutachten-Fragebogen

Bitte vollständig ausfüllen und zum Untersuchungstermin mitbringen!

NEURO-UROLOGIE

Suppl.1

1. Patientendaten

Name: …………………………………..…………………. Geburtsdatum: …………………………..

Anschrift: ..…………………………………………………………………………………………….

…………………………………………………………………………………………………

Tel. (Privat) ………………………………………… Tel. (Mobil) ……………………………………….

E-Mail: ……………………………………………………………………………………………..…

(vorheriger) Beruf ……………………………………………………………………………..…..

Unfalltag ………………………………..

Arbeitgeber zum Unfallzeitpunkt …………………………………………….................................

2. Allgemeine / Soziale Anamnese:

Körpergröße (cm) …………………….. Gewicht (kg) ………………………………

Familienstand: ……………………… Anzahl Ihrer Kinder: ………………………………

Allergien: ………………………………………………………………………………………………

……………………………………………………………………………………………………

Risikofaktoren:

Rauchen Sie? ☐ ja ☐ nein ☐ aufgehört Wenn ja, wie viel am Tag? ……… Seit wann? ….………

Alkohol? ☐ ja ☐ nein Wie viel? (z. B. 1 Flasche Bier pro Tag?) ……………………………………

3. Medikamente:

Welche Medikamente nehmen Sie ein?

Medikamentenname:	morgens	mittags	abends	nachts

Leiden Sie an Medikamentenunverträglichkeiten? ☐ Ja ☐ Nein

Wenn ja, listen Sie die Unverträglichkeiten bitte auf:

……...

………

4. Bisherige Operationen / Unfälle:

Datum	Eingriff
…………………………	……………………………………………………………………
…………………………	……………………………………………………………………
…………………………	……………………………………………………………………
…………………………	……………………………………………………………………
…………………………	……………………………………………………………………

5. Waren Sie vor dem Unfallereignis bereits schon mal in urologischer Behandlung?

Wenn ja, wann und bei welchem Facharzt für Urologie waren Sie vorstellig?

………

………

6. Vorerkrankungen:

Haben sie urologische Vorerkrankungen? Wenn ja, welche?

………

………

………

………

Welche sonstigen Vorerkrankungen / Nebenerkrankungen haben Sie?

………

………

………

………

7. Aktuelle urologische Beschwerden:

Bitte beschreiben Sie Ihre aktuellen Beschwerden im urologischen Fachbereich:

(ggf. auf gesondertem Blatt ergänzen)

Harnblase: ………………………………………………………………………………………………

………

………

………

………

………

Darm: ………

……

……

……

Sexualfunktion: ……………………………………………………………………………………………

……

……

……

8. Behandelnde Ärzte:

(Bitte Name, Adresse und Telefon- / Faxnummer angeben!)

Hausarzt: ……………………………………………………………………………………………….

…….

Urologe: ……………………………………………………………………………………………….

…….

Gynäkologe: …………………………………………………………………………………………

…….

D-Arzt: ……………………………………………………………………………………………….

…….

Weitere Ärzte: ………………………………………………………………………………………

…….

9. Schulausbildung / Berufsausbildung / Studium / Berufstätigkeit:

Welche Schulausbildung haben Sie?

Zeitraum	Schule
…………………………………………	………………………………………………………………..
…………………………………………	………………………………………………………………..
…………………………………………	………………………………………………………………..

Welche Berufsausbildung bzw. Lehrausbildung / Studium schloss sich daran an?

Zeitraum	Ausbildungsstätte
…………………………………………	………………………………………………………………..
…………………………………………	………………………………………………………………..
…………………………………………	………………………………………………………………..

Welche Berufstätigkeiten haben Sie danach ausgeübt?

Zeitraum	Tätigkeit / Arbeitgeber
…………………………………………	………………………………………………………………..
…………………………………………	………………………………………………………………..
…………………………………………	………………………………………………………………..

Welchen Beruf haben Sie zum Unfallzeitpunkt ausgeübt? Bei welcher Firma?

……….

……….

10. Rente:

EU - Rentner (Erwerbsunfähigkeit) seit wann: …………………………… wie hoch: ………………………………

BU - Rentner (Berufsunfähigkeit) seit wann: …………………………… wie hoch: ………………………………

MdE (Minderung der Erwerbsunfähigkeit) seit wann: ……………………… wie hoch: ………………………………

11. Aktuell arbeitsfähig / arbeitsunfähig:

unfallabhängig

☐ ja, bis wann: ……………………………..

☐ nein, seit wann sind Sie wieder arbeitsfähig: ……………………………..

unfallunabhängig

☐ ja, bis wann: ……………………………..

☐ nein, seit wann sind Sie wieder arbeitsfähig: ……………………………..
